# Effects of aerobic and strength-based training on metabolic health indicators in older adults

**DOI:** 10.1186/1476-511X-9-76

**Published:** 2010-07-22

**Authors:** Raul A Martins, Manuel T Veríssimo, Manuel J Coelho e Silva, Sean P Cumming, Ana M Teixeira

**Affiliations:** 1 Faculty of Sport Sciences and Physical Education, University of Coimbra, Coimbra, Portugal; 2 School of Health, University of Bath, Bath, UK

## Abstract

**Background:**

The weakening of the cardiovascular system associated with aging could be countered by increasing levels of physical activity and functional fitness. However, inconsistent findings have been found, and the variety of characteristics of exercise used in previous studies may partly explain that inconsistent results.

**Objective:**

To investigate the training effect of sixteen weeks of moderate intensity, progressive aerobic and strength-based training on metabolic health of older women and men.

**Methods:**

Sixty three sedentary individuals (mean (SD) age 76 (8) years) were randomly assigned to control (n = 31) or exercising (n = 32) groups. The training group was separated to aerobic (n = 18) or strength-based (n = 14). Training took place three times a week. Subjects agreed not to change their diet or lifestyle over the experimental period.

**Results:**

Exercising group attained after treatment significant differences on body weight, waist circumference, body mass index, diastolic blood pressure, triglycerides, total cholesterol, HDL-cholesterol, LDL-cholesterol, total cholesterol/HDL-cholesterol relationship, high sensitivity C-reactive protein, and 6-minute walk distance. The control group only had significant differences on waist circumference.

**Conclusion:**

The training programs produced significant benefits on metabolic health indicators of sedentary older women and men.

## Background

Age is associated with increases in body weight, body fat, abdominal fat [1,2], and deterioration of the lipid profile [3,4]. Time spent engaging in sedentary activities also increases with age [5,6] and is associated with obesity, atherosclerosis, and cardiovascular disease [7,8]. Conversely, regular involvement in moderate to vigorous bouts of physically active is documented as beneficial to cardiovascular health [9]. Accordingly, the weakening of the cardiovascular system associated with aging could be countered by increasing levels of physical activity and functional fitness [10,11].

Aerobic-based training has been proposed as an effective mechanism for improving cardiovascular protection, with training resulting in reductions of 2% on total cholesterol (TC), 2% on low density lipoprotein cholesterol (LDL-C) and 9% on triglycerides (TG), and increases of 3% on high density lipoprotein cholesterol (HDL-C) in men 18 years of age and older [12]. Research has also found positive training related adaptations on TC, TG, LDL-C and HDL-C [13], or only on LDL-C and TC/HDL-C, without changes on TC, HDL-C and TG [14]. However, the variety of characteristics (frequency, intensity, time, and type) of exercise used in previous studies may partly explain inconsistent findings of different modes of aerobic exercise causing unchanged TC, HDL-C or LDL-C [15,16]. Inconsistent results also exist in strength-based training programs with some failing to achieve changes in blood lipid profile in postmenopausal women [17,18] or in adult men [16], while others have reported benefits [19]. Additionally, some studies focusing on the effects of the gender in the lipid profile have found significant differences, with women having higher HDL-C, decreased LDL-C, and decreased TG comparing with men [20]. High-sensitivy C-reactive protein (hs-CRP) is known to be a sensitive marker of inflammation and has also been related to increased risk of vascular disease [21,22]. Accordingly, factors that may interfere with hs-CRP levels, such as physical activity, require further investigation in populations that are at increased cardiovascular risk.

Considering the observed deterioration of the cardiovascular system and the metabolic profile that tends to accompany aging, it is important to know the potential benefits derived from the exercise. Although the effects of aerobic versus resistance training on cardiovascular risk factors have been compared [16,23], there is a need to further investigate the effects of the aerobic-based exercise versus strength-based exercise on body weight (BW), body mass index (BMI), waist circumference (WC), lipids, blood pressure, hs-CRP, and walking distance in older women and men. Accordingly, in the current study we hypothesized that moderate intensity, progressive aerobic-based and strength-based training would improve metabolic health of older women and men.

## Results

At baseline evaluation (before treatment), no significant differences were observed between the exercising and control groups for any of the studied variables. Similarly there were no differences in the variables of interest across gender. At 16-week evaluation (after treatment), in the exercising group, no differences were observed between aerobic and strength-based training or between genders for any variable.

As shown in table 1, the exercising group attained after treatment significant differences on BW (-1%), WC (-3%), BMI (-1%), diastolic blood pressure (DBP) (-4%), TG (-11%), TC (-6%), HDL-C (5%), LDL-C (-13%), TC/HDL-C relationship (-9%), hs-CRP (-26%), and 6-minute walk distance (13%). The control group (table 2) only had significant differences on waist circumference (-2%).

**Table 1:** Exercise group

	Before	After
Body weight [kg]	73.1 (11.0)	72.3 (10.7)*
Waist circumference [cm]	93.9 (10.1)	90.5 (10.0)**
Body mass index [kg.m^-2^]	30.6 (5.0)	30.3 (4.9)*
Blood pressure [mm Hg]		
Systolic	149.1 (20.7)	149.8 (19.2)
Diastolic	76.6 (9.5)	74.2 (9.3)*
Triglycerides [mmol.L^-1^]	1.35 (0.58)	1.20 (0.54)*
Total cholesterol [mmol.L^-1^]	5.64 (0.86)	5.29 (1.03)*
HDL-cholesterol [mmol.L^-1^]	1.31 (0.25)	1.37 (0.32)*
LDL-cholesterol [mmol.L^-1^]	2.36 (0.77)	2.05 (0.86)**
Total Cholesterol/HDL-cholesterol	4.40 (0.92)	4.02 (0.81)**
hs-CRP [mg.L^-1^]	5.41 (3.94)	3.99 (1.97)*
6-minute walk distance [m]	387.0 (75.9)	437.2 (82.5)**

Values are mean (SD).

*p < 0.05, **p < 0.01 compared with before.

HDL, high-density lipoprotein; LDL, low-density lipoprotein; hs-CRP, high-sensitivity C-reactive protein.

**Table 2:** Control group

	Before	After
Body weight [kg]	70.9 (12.3)	70.4 (12.7)
Waist circumference [cm]	93.3 (9.9)	90.9 (10.2)**
Body mass index [kg.m^-2^]	29.0 (4.4)	28.8 (4.7)
Blood pressure [mm Hg]		
Systolic	146.1 (20.2)	142.5 (23.6)
Diastolic	76.2 (8.6)	75.4 (13.1)
Triglycerides [mmol.L^-1^]	1.10 (0.35)	1.15 (0.35)
Total cholesterol [mmol.L^-1^]	5.14 (0.94)	5.27 (1.02)
HDL-cholesterol [mmol.L^-1^]	1.33 (0.28)	1.32 (0.29)
LDL-cholesterol [mmol.L^-1^]	2.39 (0.86)	2.27 (0.61)
Total Cholesterol/HDL-cholesterol	3.99 (0.98)	4.07 (0.83)
hs-CRP [mg.L^-1^]	5.5 (3.5)	5.1 (2.3)
6-minute walk distance [m]	341.9 (125.9)	342.9 (169.8)

Values are mean (SD).

*p < 0.05, **p < 0.01 compared with before.

HDL, high-density lipoprotein; LDL, low-density lipoprotein; hs-CRP, high-sensitivity C-reactive protein.

At baseline, BMI correlated with TC (r = 0.35, p = 0.007), TG (r = 0.38, p = 0.004), and hs-CRP (r = 0.46, p = 0.001). WC correlated with TC (r = 0.30, p = 0.022), TG (r = 0.35, p = 0.010), hs-CRP (r = 0.38, p = 0.010), and TC/HDL-C (r = 0.38, p = 0.005). Finally, BW also correlated with TC (r = 0.33, p = 0.011), TG (r = 0.27, p = 0.044), hs-CRP (r = 0.40, p = 0.006), and TC/HDL-C (r = 0.33, p = 0.016).

## Discussion

The main finding of this study is that both exercise programs (i.e., aerobic and strength-based) resulted in positive changes in important cardiovascular risk factors, namely TC, TG, LDL-C, HDL-C, TC/HDL-C, hs-CRP, DBP, BMI, WC, and BW, in a previously sedentary group of older women and men. Moreover, cardiorespiratory fitness also improves as result of the increase on the 6-minute walk distance. Dietary composition is a potentially confounding factor and is known to influence lipoprotein concentrations. To minimize any confounding effects associated with variation in diet, all participants were fed a similar diet during the 16 weeks of the investigation. Both programs were successfully in that they both resulted in 13% gains on 6-min walk distance. This observation suggests that aerobic and strength based exercise programs may result in improved cardiovascular functionality in older participants, potentially countering the documented age-related decline in peak oxygen uptake.

Following studies examining the effects of endurance and strength training on cardiovascular health have generally found either positive changes in lipid profile or no changes at all. More favorable changes in response to training occur usually in those with more pronounced dyslipidemia at baseline [24], and have been pointed as dependent on loss of body fat [25]. Our baseline values were normal for TG and HDL-C, optimal for LDL-C, and desirable (control group) and borderline high (exercising group) for TC, according to the guidelines [26]. Also, in the current study, at baseline in the total participant group, BMI, WC and BW correlated positively with TC, TG and hs-CRP. Moreover, WC and BW correlated with TC/HDL-C.

Our finding that TC, LDL-C, TG, and TC/HDL-C diminish with exercise is consistent with previous research [12,13], even those programs that have not attained resulted in gains after strength training in postmenopausal women [17] and in adult men [16]. To explain why some programs have not resulted in gains, some researchers have pointed a dose-response relationship between serum lipid levels (TC, TG, HDL-C, and TC/HDL-C) and levels of physical activity (intensity and duration) in adult women and men [6]. Accordingly, Cox and colleagues [27] have demonstrated lower TC and LDL-C after 6 months of higher-, but not lower-, intensity exercise in middle and older sedentary women. However, Sillanpaa and colleagues [16] failed to attain changes on TC, TG and LDL-C after 21-week of high-intensity endurance and heavy resistance strength training in healthy 40-65-year-old men. Independent of the mechanism underlying lipid changes, a reduction of 1% on TC has been shown to reduce the risk for coronary artery disease by 2% [28], which implies that our exercising participants have reduced about 12% their risk. Moreover, a 1% reduction in LDL-C reduces the risk of major coronary events by approximately 2% [29], which means that we have about a 26% gain.

Our finding that HDL-C increases with exercise is consistent with previous results [12,13,19]. However, not all studies have found gains in HDL-C following aerobic-based [14-16] or strength-based programs [16-18]. This absence of gains have been justified with the higher initial levels of HDL-C [25,30], with the lower exercise intensity [31], with the good initial body composition [12], and with the lack of control of the time of blood sampling [32]. Seasonal fluctuations [33] and changes in dietary fat intake or leisure time physical activity may also influence the changes in the lipid profile [34,35]. Our baseline HDL-C values were within normal range, and both exercising programs seem to have had enough intensity to promote gains on HDL-C, accompanied by reduction on BW, BMI, and WC. Also, a gap of at least 48 hours occurs between the last training session and the sampling. As a decrease of 1% on HDL-C has been associated with a 2-3% increase in the risk for coronary heart disease (CHD) [36], and assuming that the reverse is true, the 5% increase observed in our both programs should decrease CHD by 10-15%.

Recent data have shown decreases on resting blood pressure after prolonged endurance training of 1.9/1.6 (SBP/DBP) mmHg [37], and after strength training of about 3 mmHg in both SBP and DBP [38,39]. This positive effects caused by the physical training have been more pronounced among hypertensive participants [39]. Participants in this study had normal DBP (<80 mm Hg) and high SBP (140-159 mm Hg) at baseline. Interestingly, after the training only DBP have reduced 3 mm Hg that may have clinical and biological relevance in the risk of heart disease.

In this study, aerobic and strength-based training have reduced 26% inflammation as measured by hs-CRP concentrations. Previous findings on the effect of strength and endurance training on hs-CRP levels have been inconsistent, with some studies showing benefits [40,41] and others showing no effect at all [42,43]. The mechanisms underlying an exercise training-induced reduction in serum hs-CRP concentrations remain unknown. However, the lack of effects of exercise on hs-CRP has been explained by the few concomitant metabolic risk factors of the participants [16], and by a lack of changes in anthropometric variables (e.g., body weight, waist circumference) [43]. In fact, our participants at baseline have been sedentary, obese - particularly the exercising group (mean (SD) BMI 30.6 (5.0) kg/m^2^), and hypertensive (mean (SD) SBP 148 (20) mmHg). Moreover, the changes on BMI, WC and BW could help to justify the changes on hs-CRP. Additionally, the gains on muscular function as result of the increases on 6-minute walk distance would also influence the hs-CRP.

In conclusion, the training programs used in this study produced significant benefits on 6-minute walk distance, DBP, BW, WC, BMI, TG, TC, HDL-C, LDL-C, TC/HDL-C, and hs-CRP. Accordingly, the results of the current study suggest that moderate intensity aerobic-based and strength-based programs, with 16 weeks of duration, are enough to positively influence the metabolic health indicators of sedentary older women and men.

## Methods

### Participants

Sixty three sedentary individuals (65-95 years old) volunteered (mean (SD) age 76 (8) years) to participate in this study (38 women, 25 men). Participants were informed about any potential risks and/or discomforts associated with participation in the study and were required to provide their written informed consent before being included in the study. Participants who fulfilled the inclusion criteria and passed the baseline physical examination were randomized into two training groups and one control group. All the participants were from the same institution (St. House of Charity) and were provided with similar diets, in terms of caloric intake and nutrients, controlled by a nutritionist. Participants who were taking medications including aspirin and statins maintained unaltered posologies during the study. All the participants in the training groups performed the training as planned. The work was a part of a larger project, and the data have been partially reported earlier with a smaller number of subjects [44]. The study conforms to the laws of the country in which took place, and was approved by an ethical review board at University of Coimbra.

### Exclusion criteria

Participants with impaired glucose tolerance and diabetes were excluded. All physical or psychological diseases that may have precluded ability to perform the requested training exercises and testing, and medications know to influence physical performance or interpretation of the findings were also considered exclusion criteria.

### Aerobic-based training

The training was supervised by an Exercise Physiologist, and the frequency was kept three times per week for 16 weeks, with 45 minutes per session. The intensity of the main part of the session started with work heart rate (HR) of 40-50%HR_reserve _(1-4^th ^week), increasing progressively to 51-60%HR_reserve _(5-8^th ^week), 61-70%HR_reserve _(9-12^th ^week), and to 71-85%HR_reserve _(13-16^th ^week).

### Strength-based training

After an adequate warm up, the participants completed resistance exercise for three days a week for 16 weeks. They performed eight exercises with elastic bands for the major muscular groups respecting the following progression: 1 set of 8 repetitions (1-2^nd ^week), 1 set of 12 reps (3-4^th ^weeks), 2 sets of 8 reps (5-6^th ^weeks), 2 sets of 10 reps (7-8^th ^weeks), 2 sets of 12 reps (9-10^th ^week), 2 sets of 15 reps (11-12^th ^weeks), 3 sets of 12 reps (13-14^th ^weeks), and 3 sets of 15 reps (15-16^th ^weeks). An interval period of at least three minutes was assured between sets of the same exercise.

### Anthropometry

Anthropometric assessment was carried out in a separate room, taking care to ensure the participants privacy. Body weight was determined using a portable scale (Seca^®^, model 770, Germany) with a precision of 0.1 kilogram. Waist circumference was measured using a retractable glass fiber tape measure (Hoechstmass-Rollfix^®^, Germany) with a precision of 0.1 centimeters. Stature was determined using a portable stadiometer (Seca Bodymeter^®^, model 208, Germany) with a precision of 0.1 centimetres.

### Functional fitness

The *6-minute walk test *was applied before and after exercising programs and assessed the aerobic endurance measuring the distance around a 50 metres course covered in 6-minutes [45].

### Blood pressure

Resting blood pressure was measured by the auscultation method using sphygmomanometer (Aneroid Sphygmomanometer-HICO HM 1001^®^, Germany) and stethoscope (Nurse Type Professional Stethoscope-HICO HM-3005^®^, Germany). Participants were in sitting position respecting the procedures for assessment of resting blood pressure presented by the ACSM [46].

### Blood sampling

Venous blood samples were collected into EDTA containing tubes, in the morning between 8:00 am and 9:30 am by two specialized nurses, after 12 hours fasting, and after a minimum of 48 hours since the last physical exercise intervention. Participants were in a seated position and rested for five minutes. Determinations of the lipids and hs-CRP used a bench top clinical chemistry analyzer (RX imola, Randox Laboratories Ltd, UK). Determination of hs-CRP concentration was done by immunoturbidimetry (High sensitivity CRP kit, Randox Laboratories Ltd, UK). All the cholesterol determination tests used were direct enzymatic clearance tests from the Randox Laboratories. TC was determined using a Trider-based (CHOD-PAP) colorimetric end-point assay (CH 3810, Randox Laboratories Ltd, UK). HDL-C was determined using a direct two-point kinetic assay kit (CH 2652, Randox Laboratories Ltd, UK). LDL-C determination was done using a direct two-point kinetic assay kit (CH 9702, Randox Laboratories Ltd, UK). TG were determined using a Trinder-based (GPO-PAP) colorimetric end point assay (TR 3823, Randox Laboratories Ltd, UK). All the methods were controlled and validated using external controls from INSA and RIQAS.

### **Statistical analysis**

Differences between groups (exercising and control), between genders (females and males), and between exercising subgroups (aerobic and strength-based) were analyzed using a univariate analysis of variance. Differences between evaluations (before and after) were analyzed with a univariate analysis of variance for repeated measures. F and p values from the Pillai test were calculated for within participant's comparisons. Bonferroni's tests were used for multiple comparisons. Values are expressed as mean and standard deviation (SD), and statistical significance was accepted at the p < 0.05 level.

## Competing interests

The authors declare that they have no competing interests.

## Authors' contributions

RAM participated in the design of the study, participated in the exercise protocols, performed the statistical analysis and drafted the manuscript. MTV, MJCS and AMT participated in the design of the study and in its coordination and helped to draft the manuscript. SPC participated in the draft of the manuscript and revising it critically for important intellectual content. All authors read and approved the final manuscript.
